# Evaluating the Preliminary Effectiveness of the Person-Centered Care Assessment Tool (PCC-AT) in Zambian Health Facilities: Protocol for a Mixed Methods Cross-Sectional Study

**DOI:** 10.2196/54129

**Published:** 2024-07-23

**Authors:** Jessica Posner, Adamson Paxon Ndhlovu, Jemmy Mushinka Musangulule, Malia Duffy, Amy Casella, Caitlin Madevu-Matson, Nicole Davis, Melissa Sharer

**Affiliations:** 1 International Division JSI Arlington, VA United States; 2 Health Across Humanity, LLC Boston, MA United States; 3 Department of Public Health St. Ambrose University Davenport, IA United States

**Keywords:** person-centered care, HIV, action plans, preliminary, person-centered care assessment tool, PCC-AT, assessment tool, Zambia, health facility, exploratory study, HIV treatment, inequities, framework, practitioners, health services

## Abstract

**Background:**

Person-centered care (PCC) within HIV treatment services has demonstrated potential to overcome inequities in HIV service access while improving treatment outcomes. Despite PCC being widely considered a best practice, no consensus exists on its assessment and measurement. This study in Zambia builds upon previous research that informed development of a framework for PCC and a PCC assessment tool (PCC-AT).

**Objective:**

This mixed methods study aims to examine the preliminary effectiveness of the PCC-AT through assessing the association between client HIV service delivery indicators and facility PCC-AT scores. We hypothesize that facilities with higher PCC-AT scores will demonstrate more favorable HIV treatment continuity, viral load (VL) coverage, and viral suppression in comparison to those of facilities with lower PCC-AT scores.

**Methods:**

We will implement the PCC-AT at 30 randomly selected health facilities in the Copperbelt and Central provinces of Zambia. For each study facility, data will be gathered from 3 sources: (1) PCC-AT scores, (2) PCC-AT action plans, and (3) facility characteristics, along with service delivery data. Quantitative analysis, using STATA, will include descriptive statistics on the PCC-AT results stratified by facility characteristics. Cross-tabulations and/or regression analysis will be used to determine associations between scores and treatment continuity, VL coverage, and/or viral suppression. Qualitative data will be collected via action planning, with detailed notes collected and recorded into an action plan template. Descriptive coding and emerging themes will be analyzed with NVivo software.

**Results:**

As of May 2024, we enrolled 29 facilities in the study and data analysis from the key informant interviews is currently underway. Results are expected to be published by September 2024.

**Conclusions:**

Assessment and measurement of PCC within HIV treatment settings is a novel approach that offers HIV treatment practitioners the opportunity to examine their services and identify actions to improve PCC performance. Study results and the PCC-AT will be broadly disseminated for use among all project sites in Zambia as well as other HIV treatment programs, in addition to making the PCC-AT publicly available to global HIV practitioners.

**International Registered Report Identifier (IRRID):**

DERR1-10.2196/54129

## Introduction

### Background

Given that the HIV epidemic in Zambia is largely concentrated among women and key population members who are highly marginalized, person-centered care (PCC) is perceived as a priority to advance outcomes for people living with HIV [1-4]. The World Health Organization [5] describes a person-centered framework as one in which:

all people have equal access to quality health services that are co-produced in a way that meets their life course needs and respects their preferences, are coordinated across the continuum of care and are comprehensive, safe, effective, timely, efficient, and acceptable and all carers are motivated, skilled and operate in a supportive environment.

From a health systems perspective, PCC can improve job satisfaction, reduce overall costs, and increase efficiencies and resilience [6]. From a client perspective, PCC models are increasingly recognized as critical to overcome inequities in health service access while increasing participation in quality and responsive care, improving health literacy and self-care, and increasing satisfaction with care [7,8].

The Joint United Nations Programme on HIV/AIDS (UNAIDS) reported that the potential benefits of PCC for clients extend to improved clinical outcomes [7]. A study in South Africa demonstrated that person-centered approaches such as 3-month community-based differentiated antiretroviral treatment (ART) service delivery resulted in improved client retention over the standard of care [9]. In Uganda, monthly support groups for adolescents and parents/caregivers, telephone appointment reminders, and notification of missed visits contributed to increased retention in care [10]. In Zimbabwe, community adolescent treatment supporters provided monthly support and ART refill groups for adolescents, cofacilitated caregiver workshops, provided individual health education and counseling, sent SMS text message reminders and check-ins, provided home visits, and referred and linked adolescents to care, leading to significant increases in ART initiation, viral suppression, and ART adherence [11,12]. In Mozambique, a combination intervention approach including SMS text message appointment reminders and individualized health messages contributed to improved linkage to care [13].

In Zambia, 89% of HIV-positive adults (aged 15+ years) are aware of their status, 97% of adults diagnosed with HIV report being on ART, and 96% of those on ART are virally suppressed [4]. At 11.7%, the HIV prevalence has been steadily declining from a high of 14% in 2000 [14]. However, progress in this regard is not universal. The national HIV prevalence rate is estimated to be 49% among female sex workers [14], 22% among transgender people, 21% among men who have sex with men, 15% among people who inject drugs, and 14%-27% among people who are incarcerated [1]. Although adolescents and young people (aged 15-24 years) represent 20% of the total population of Zambia, they account for 40% of all new infections [15], and the HIV prevalence among adolescent girls and young women is double that of adolescent boys and young men [16]. Among these key and priority populations, elevated HIV risk is associated with multiple determinants, including criminalization, stigma and discrimination, harmful gender norms, and limited access to acceptable health services. To advance progress for the most vulnerable populations, person-centered HIV treatment service delivery models that are tailored to the needs and expectations of diverse populations offer an important opportunity to help overcome service access, uptake, and continuity challenges.

Despite PCC being widely considered a best practice in serving both the needs of recipients of care and the goals of reaching epidemic control more broadly, no consensus exists on how to assess and measure the construct. This study conducted in Zambia will build upon previous evidence collected by the study team, including (1) a systematic literature review, which further defined the construct of PCC and led to a proposed framework that informed the PCC assessment tool (PCC-AT) [17]; and (2) a PCC-AT content validity, score consistency and reliability, and feasibility study conducted in Ghana [18].

**Figure 1:**
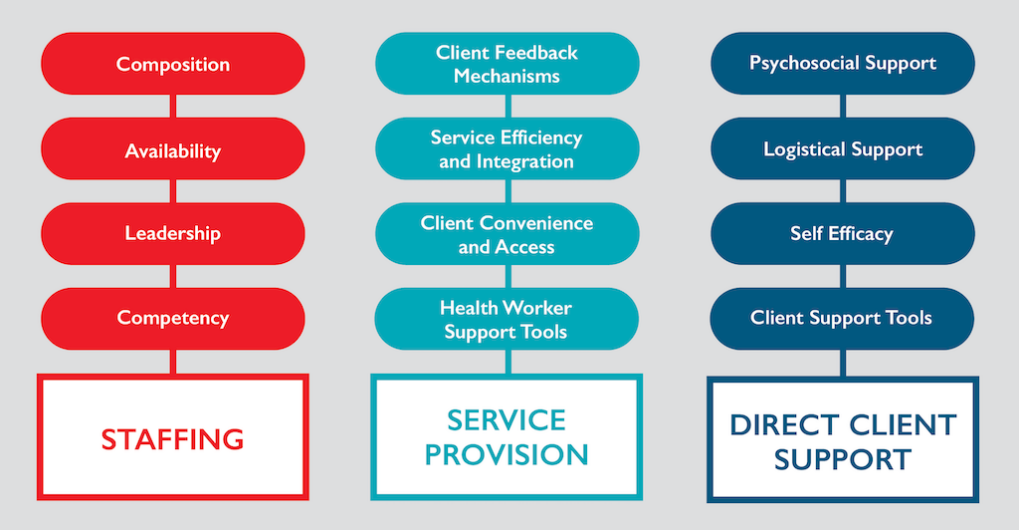
Domains and subdomains of the person-centered care assessment tool.

The PCC-AT is an Excel-based tool that measures health facility staff perspectives on PCC service delivery within HIV treatment settings. Implementation of the PCC-AT requires in-depth discussions among health facility staff to assign a score using a benchmarking approach (eg, simple yes/no or mostly/partly/none responses) for each of the 55 performance expectations linked to 3 domains and 12 subdomains (see Figure 1). Similar to other tools that are widely used to measure the organizational and technical performance of implementing partners, the PCC-AT includes dropdown menus and formulas to calculate composite scores from multiple tabs. The PCC-AT culminates in an “action planning” process, a template for which is embedded within the tool itself, to design tailored interventions and improvements based on PCC-AT scores.

### Objectives and Study Questions

This study seeks to examine the correlation between PCC-AT scores with HIV service delivery indicators, including treatment continuity, viral load (VL) coverage, and viral suppression, at 30 randomly selected health facilities in the Copperbelt and Central provinces of Zambia. This study also seeks to identify the types of PCC-strengthening activities that health facility staff identify as priorities based on PCC-AT scores. Specifically, we aim to answer the following research questions: (1) Do facilities with higher PCC-AT scores demonstrate higher performance of HIV service delivery indicators in comparison to facilities with lower PCC-AT scores? and (2) What common and unique themes emerge from PCC action planning in facilities with high PCC-AT scores compared with facilities with low PCC-AT scores? We hypothesize that facilities with higher PCC-AT scores will demonstrate more favorable HIV treatment continuity, VL coverage, and viral suppression in comparison to those of facilities with lower PCC-AT scores.

## Methods

### Study Design

This research is based on a mixed methods, cross-sectional study design, and will be guided by a vulnerable and marginalized populations approach [19] under a health equity lens [20].

### Site Selection and Study Setting

The study will be carried out at USAID DISCOVER-Health project sites (N=66) in the Copperbelt and Central provinces, which are public, primary-level facilities. Sites will be considered eligible for inclusion if the health facility (1) provides direct HIV treatment services and (2) expresses interest in scaling PCC to strengthen services. Prisons associated with health facilities are excluded from randomization (n=9) due to differences in how services are delivered at such sites. The 57 eligible health facilities are spread across urban, periurban, and rural locations; hence, the study’s sample frame is based on a proportionate stratification method to stratify the health facilities by rural, periurban, and urban locations. All sites are considered primary sites. Using a random-number generator in Excel, 30 health facilities were selected as data collection sites from the sampling frame to ensure that selected clinics have a proportionate representation of urban, periurban, and rural facilities to more precisely reflect the range of facility characteristics in the overall sample frame. As needed, alternate facilities will be selected from the randomly ranked stratum lists in ascending order (see Multimedia Appendix 1).

### Participants

At each randomly selected health facility, 5-10 English-speaking health facility staff members, based on availability, will be selected to participate in the PCC-AT on the day of data collection. Inclusion criteria for participants include at least 1 year of employment or volunteering at the study health facility and direct provision of or knowledge related to HIV treatment services (eg, ART providers, nurses, lab technicians, counselors, pharmacists [or the equivalent], data managers, and community group liaisons). Where there are more than 7 eligible staff members, participation of staff based on a gender balance and experience will be encouraged. Exclusion criteria for participation in the PCC-AT study include any staff who have worked or volunteered at the study facility for less than 1 year and any staff member whose job description does not include direct HIV treatment service delivery or knowledge of how services are delivered.

### Study Procedure

#### PCC-AT Implementation

PCC-AT implementation at the health facilities will be conducted by full-time English-speaking project staff members, divided into 6 data collection teams with each team composed of 3 data collectors. All data collectors will undergo a 2-day training led by the PCC-AT developers, consisting of a general introduction on the purpose and nature of the study, along with instruction on obtaining informed consent, interviewing and facilitation techniques, data storage, and practice implementing the PCC-AT.

Each data collection team will designate a lead facilitator responsible for introducing the study, collecting informed consent forms, and facilitating the PCC-AT process with the health facility team through a guided discussion. The lead facilitator will guide PCC-AT implementation in a neutral manner so that health facility staff assess their own strengths and weaknesses. The assessment process is intended to promote information sharing, a healthy internal dialogue, and consensus building by including a range of staff who will discuss institutional abilities, systems, procedures, and policies in various domains that influence PCC. The goal is to arrive at a consensus for the 55 performance expectations across each of the 12 subdomains of the PCC-AT.

#### Action Planning

Once the scoring is complete, each PCC-AT implementation will culminate in an action-planning process. The lead facilitator will (1) guide health facility staff to identify low scores on the PCC-AT; (2) lead an examination of the performance expectations to identify why the facility had a low score; and (3) facilitate a discussion so that health facility staff identify potential solutions, rank the potential solutions in order of their ability to be implemented by the staff, and identify the priority activities and a timeline for action. The remaining study team members will take detailed notes that document the discussion on an action planning template to capture the full universe of potential activities and the rationale behind activity selection. It is anticipated that PCC-AT implementation, including action planning, will require approximately 2 hours, taking place in the afternoon when patient flow is light in a private room located within each health facility.

#### Service Delivery Data Collection

Following PCC-AT implementation, the study team will select key indicators within the last 3 months (April 1 to June 30, 2023) from each of the 30 selected health facilities, in alignment with the timing of the PCC-AT study activities. This study will use the monitoring, evaluation, and reporting guideline of the President’s Emergency Plan for AIDS Relief version 2.6 definitions and calculations [21] for each indicator of interest, including (1) continuity of treatment, (2) VL coverage, and (3) viral suppression.

Continuity of treatment is a proxy measure of the overall gain or loss in patients compared to the expected number of patients on treatment, which is calculated by tracking the number of adults and children currently receiving ART (TX_CURR) over time to measure the ongoing scale-up and uptake of ART and continuity of treatment in ART programs.

VL coverage is a comparison of the number of patients on ART with a VL result documented in the medical or laboratory records/laboratory information system (LIS) within the past 12 months (viral suppression indicator denominator) with the result for TX_CURR from 6 months earlier.

Viral suppression reflects the percentage of patients on ART with a suppressed VL result (<1000 copies/mL) documented in the medical or laboratory records/LIS within the past 12 months as the numerator and the total number of patients on ART with a VL result documented in the medical or laboratory records/LIS within the past 12 months as the denominator. This indicator monitors the proportion of documented VL results from adult and pediatric ART patients who have been on ART for at least 3 months (or according to national guidelines) with a suppressed VL result (<1000 copies/mL). This allows ART programs to monitor the individual and overall programmatic response to ART as measured by virologic suppression.

### Data Analysis

#### Overall Analysis Plan

Data analysis will include all 3 sources, (1) the PCC-AT composite score page, (2) PCC-AT action plan template, and (3) descriptive facility statistics (eg, client population size, urban/periurban/rural location, and province), as well as the service delivery data for these 3 key indicators.

#### Quantitative Data

We will investigate if a correlation exists between the 3 key indicators of interest, including treatment continuity, VL coverage, and viral suppression, and PCC-AT domain/subdomain scores using STATA or similar software. Analysis will include descriptive statistics (mean, median, range) on the PCC-AT results by domain and subdomain while stratifying by facility characteristics. In addition, cross-tabulations and/or regression analysis will be performed to determine if there are any associations between scores and treatment continuity, VL coverage, and/or viral suppression. 

#### Qualitative Analysis

Qualitative data from the action planning template will be separately analyzed using the detailed notes in the template. These notes will be analyzed via NVivo or similar software. Axial and open coding of the action plan text will allow for deconstruction of the text and uncovering of emergent themes, concepts, issues, and ideas. Action plans will be coded separately by 2 members of the study team according to the framework and compared to ensure interrater reliability.

### Materials

In addition to the PCC-AT, materials will include the study information sheet and consent form (Multimedia Appendix 2) and the PCC-AT action planning template (Multimedia Appendix 3). All PCC-AT activities will be recorded and take place in English.

### Ethical Considerations

The study received ethical approval from the Zambia National Health Research Authority (reference number 4150-2023) and was determined to be exempt from human subjects’ oversight by the JSI Institutional Review Board (23-36E). Facilitators will be trained in informed consent and confidentiality principles and protocols. All study participants will be asked if they are interested in participating in the study. Study participants will be presented with a study information sheet and a consent form in English, which they will be asked to sign if they agree to participate in the study. The consent form explicitly states that participation is voluntary. In-kind provisions (ie, water, snacks, lunch) will be provided depending on the length of each session. There will be no monetary compensation for participation. Refusal to participate will have no bearing on health providers’ work assignment in the future or payments for health work. Participants may choose to stop at any point during the interview or discussion with no penalty or bearing on their employment status. Only the study team will have access to data. Participant confidentiality will be protected throughout study procedures, including (1) excluding the names of participants on all data collection materials; (2) storing data in a secure place; (3) developing codes during qualitative data analysis so that any potential identifiers (eg, staff positions) are not associated with the data; and (4) maintaining strict adherence to the principles of voluntary participation, confidentiality, anonymity, and protection of human subjects as guaranteed by the consent form (Multimedia Appendix 2).

## Results

Data collection in Zambia took place in July and August 2023 and data analysis was performed in September-November 2023. One manuscript related to this work has been published [18]. As of May 2024, we enrolled 29 facilities in the study and data analysis from the key informant interviews is currently underway. All results are expected to be published by September 2024.

## Discussion

This study in Zambia seeks to understand associations between PCC-AT scores and HIV clinical outcomes, contributing to the overall evidence base on the importance of a person-centered approach to delivering HIV care. Specifically, the data from this study will increase the existing evidence on how to measure, evaluate, and implement PCC at the primary health care level in the Zambian context. PCC has demonstrated potential to contribute to health equity for vulnerable and/or marginalized populations [22]. Given that the HIV epidemic in Zambia is largely concentrated among vulnerable and marginalized populations, PCC is a critical measure to overcome HIV service access limitations and to advance health outcomes [4,23].

A potential limitation of this study is the high performance of the majority of health facilities included in the sample frame for each of the indicators of interest. Given that the majority of clients are retained on treatment, have recent VLs (within the past 12 months), and have achieved viral suppression, it will be challenging to detect differences in service delivery indicators between groups with high and low PCC-AT scores. In addition, the PCC-AT has not yet been validated; results from this study will provide information on initial effectiveness, which will guide priorities for a future validation study. The PCC-AT also does not measure external factors that may influence scores and external generalizability is limited, although the clinic randomization process improves our ability to generalize results to other Zambian HIV treatment facilities.

These study results will inform any required refinements to the PCC-AT so that it more effectively measures PCC as identified through treatment continuity, VL coverage, and viral suppression. Assessment and measurement of PCC within HIV treatment settings is an approach that can offer HIV treatment practitioners the opportunity to critically examine their services, identify actions to improve PCC performance, and ultimately improve HIV treatment outcomes for vulnerable populations. Given the role that PCC has demonstrated to contribute to improving HIV treatment outcomes [24,25] and the potential for the PCC-AT to contribute to strengthened person-centered HIV treatment services, the study team anticipates publishing study findings in 2024 and presenting the findings at the International Conference on AIDS and STIs in Africa, which may include a training session for global health professionals in use of the tool.

After data are analyzed and if positive results emerge, it is anticipated that the PCC-AT will be broadly disseminated for use among all 173 DISCOVER-Health health facilities in Zambia as well as other HIV treatment programs in Ghana supported by this study team. This tool and process may improve PCC, and could be a valuable, strengths-based person-centered tool for the global HIV community with the aim of strengthening HIV treatment outcomes for all people.

## Appendix

Multimedia Appendix 1 Randomly identified study facilities and alternate sites.

Multimedia Appendix 2 Participant and consent forms.

Multimedia Appendix 3 Action plan template.

## Abbreviations

ART: antiretroviral treatment
LIS: laboratory information system
PCC: person-centered care
PCC-AT: person-centered care assessment tool
TX_CURR: number of adults and children currently receiving antiretroviral treatment
UNAIDS: The Joint United Nations Programme on HIV/AIDS
VL: viral load
