# Supplementary material for: Evaluating the Preliminary Effectiveness of the Person-Centered Care Assessment Tool (PCC-AT) in Zambian Health Facilities: Protocol for a Mixed Methods Cross-Sectional Study
Source: JMIR Res Protoc. 2024 Jul 23;13:e54129. doi: 10.2196/54129 (PMC11303880; doi:10.2196/54129)
Supplement: Multimedia Appendix 1 [file resprot_v13i1e54129_app1.docx]

**Multimedia Appendix 1. List of Randomly Identified Study Facilities and Alternates**

**Peri-Urban Sample**

| **Ranking** | **Name of facility** | **District** | **Province** |
| --- | --- | --- | --- |
| **1** | Waya | KABWE | CENTRAL |
| **2** | Magandanyama HP | KABWE | CENTRAL |
| **3** | Baluba Pole line | LUANSHYA | COPPERBELT |
| **4** | Fitobaula | CHILILABOMBWE | COPPERBELT |
| **5** | Lyobeka | MPONGWE | COPPERBELT |
| **6** | Matilyo | KAPIRI MPOSHI | CENTRAL |
| **Alternate** | | | |
| **7** | Mutaba RHC | KAPIRI MPOSHI | CENTRAL |
| **8** | Kalengwa South | KALULUSHI | COPPERBELT |
| **9** | Mulundu | MKUSHI | CENTRAL |
| **10** | Mwanjuni | CHIBOMBO | CENTRAL |
| **11** | Musenga | CHINGOLA | COPPERBELT |

*Sample calculation: (sample size/population size) × stratum size = 30/57 x 11 = 6

**Rural Sample**

| **Ranking** | **Name of facility** | **District** | **Province** |
| --- | --- | --- | --- |
| **1** | Chilumba | KAPIRI MPOSHI | CENTRAL |
| **2** | Chamakubi | CHIBOMBO | CENTRAL |
| **3** | Tuyu | LUANO | CENTRAL |
| **4** | Kachele | CHISAMBA | CENTRAL |
| **5** | Kaseba | SERENJE | CENTRAL |
| **6** | Kakulu | KAPIRI MPOSHI | CENTRAL |
| **7** | Kanona | SERENJE | CENTRAL |
| **8** | Namayani | CHIBOMBO | CENTRAL |
| **9** | Likumbi RHC | KAPIRI MPOSHI | CENTRAL |
| **Alternate** | | | |
| **10** | Lunchu B HP | KAPIRI MPOSHI | CENTRAL |
| **11** | Kapopo | CHIBOMBO | CENTRAL |
| **12** | Chabona HP | CHIBOMBO | CENTRAL |
| **13** | Twatasha | MKUSHI | CENTRAL |
| **14** | Kasokota | MKUSHI | CENTRAL |
| **15** | Golden Valley HP | CHIBOMBO | CENTRAL |
| **16** | Keembe Ranch | CHIBOMBO | CENTRAL |
| **17** | Mfulabunga | MPONGWE | COPPERBELT |
| **18** | Itumbwe Health Post | CHIBOMBO | CENTRAL |

*Sample calculation: (sample size/population size) × stratum size = 30/57 x 18 = 9

**Urban Sample**

| **Ranking** | **Name of facility** | **District** | **Province** |
| --- | --- | --- | --- |
| **1** | SDA_Buchi | KITWE | COPPERBELT |
| **2** | Peter Singogo | NDOLA | COPPERBELT |
| **3** | SDA_Chibolya | MUFULIRA | COPPERBELT |
| **4** | TVTC Luanshya | LUANSHYA | COPPERBELT |
| **5** | Kantolomba | NDOLA | COPPERBELT |
| **6** | Kasumbalesa | CHILILABOMBWE | COPPERBELT |
| **7** | Twaiteka Health Post | KALULUSHI | COPPERBELT |
| **8** | East of Garneton | KITWE | COPPERBELT |
| **9** | Chipulukusu_SDA | NDOLA | COPPERBELT |
| **10** | Mushili Old Clinic | NDOLA | COPPERBELT |
| **11** | UCZ_Kaloko | NDOLA | COPPERBELT |
| **12** | Katondo | NDOLA | COPPERBELT |
| **13** | SDA_Chamboli | KITWE | COPPERBELT |
| **14** | Konkola | CHILILABOMBWE | COPPERBELT |
| **15** | Nkwazi | NDOLA | COPPERBELT |
| **Alternate** | | | |
| **16** | Central Police_Kitwe | KITWE | COPPERBELT |
| **17** | UCZ_Mulenga | KITWE | COPPERBELT |
| **18** | Kings | CHINGOLA | COPPERBELT |
| **19** | Sitwe Health Post | KALULUSHI | COPPERBELT |
| **20** | Allessandras Urban Health Centre | LUANSHYA | COPPERBELT |
| **21** | Kawama Luanshya | LUANSHYA | COPPERBELT |
| **22** | SDA_Ipusukilo | KITWE | COPPERBELT |
| **23** | Chreso Ministries_Kabwe | KABWE | CENTRAL |
| **24** | Lulamba Health Post | CHINGOLA | COPPERBELT |
| **25** | Kabangwe | CHIBOMBO | CENTRAL |
| **26** | Town/Centre | NDOLA | COPPERBELT |
| **27** | UCZ_Twatasha | KITWE | COPPERBELT |
| **28** | UCZ_Kawama | KITWE | COPPERBELT |

*Sample calculation: (sample size/population size) × stratum size = 30/57 x 28 = 15
