# Supplementary material for: Evaluating the Preliminary Effectiveness of the Person-Centered Care Assessment Tool (PCC-AT) in Zambian Health Facilities: Protocol for a Mixed Methods Cross-Sectional Study
Source: JMIR Res Protoc. 2024 Jul 23;13:e54129. doi: 10.2196/54129 (PMC11303880; doi:10.2196/54129)
Supplement: Multimedia Appendix 2 [file resprot_v13i1e54129_app2.docx]

**Multimedia Appendix 2: Informed Consent**

**Informed Consent for Health Providers/Managers**

**Study Title:** **Evaluating the Preliminary Effectiveness of the Person-Centered Care Assessment Tool (PCC-AT) in Zambian Health Facilities**

Greetings. My name is __________________. We are here for the USAID DISCOVER-Health project for on behalf of JSI Research & Training the Zambian Ministry of Health. JSI has developed a tool to measure person-centered care (PCC) in facility settings to improve PCC in HIV service delivery programs. Person-centered care (PCC) is a component of ‘quality of care’ that moves beyond clinical quality of care to include concepts such as support, respect, and autonomy.

We are interested in working with you to refine the tool to improve the ability of facilities to deliver PCC for HIV services delivery and treatment. We would like your opinions and experiences providing PCC services to your clientele. As part of the study, you will participate in a guided discussion to complete the tool with fellow staff members. The questions will help us gain a deeper understanding of your experiences and challenges in provide PCC in HIV treatment. The discussion will take approximately 2 hours. Your answers will be used to help us improve the tool and framework to better measure PCC and serve as a guide for HIV treatment centers in Zambia in the future.

All information obtained from you, including session recordings, will be kept strictly confidential. No one outside the research team will be given access to any of your information. We will not use your name in any report. Additionally, your name will not be used/recorded in any recording, note or papers. Instead, we will use codes. Once information that identifies you has been removed, the remaining information you provide may be shared publicly or with third parties, without additional informed consent from you or your legal representative.

Please know that the decision to participate in this interview is completely voluntary and that your decision will not affect our employment. You have the right to skip or refuse to answer any questions you do not feel comfortable answering. You also have the right to discontinue the interview at any point. There is no penalty for refusing to participate, now or in the future.

The discussion will be guided by an external facilitator and the team will note your responses. We will be recording the tool administration process and discussion so that we do not miss any of the important points raised. However, if you choose not to be recorded, we will only take notes instead.

You will receive no direct benefit from your participation in this study. However, your participation may help improve your understanding of how to provide person-centered care to your clients and provide information to improve your facility’s performance overall. There are no known risks to being involved in the study.

In case you need any more information about the study, you may contact the Principal Investigator: **Adamson Paxon NDHLOVU, +260963674731, adamson_ndhlovu@zm.jsi.com**

Do you have any questions about the study? Do I have your agreement to proceed and agreement to be recorded?

**PARTICIPANTS’ STATEMENT**

I acknowledge that I have read or have had the purpose and contents of the Informed Consent Sheet read and all questions satisfactorily explained to me in a language I understand (……*name of language*). I fully understand the contents and any potential implications as well as my right to change my mind (i.e., withdraw from the research) even after I have signed this form.

I voluntarily agree to be part of this research and consent to being recorded.

Name of Participant…………………………………………………………………………………………………………………………....

Participants’ Signature ………………………………………………………………………………………………………….…………...

OR Thumb Print…………………………………………………………………………………………………………………………….……

Date: …………………………………………………………………………………………………………………………………………..…….
