# Supplementary material for: Evaluating the Preliminary Effectiveness of the Person-Centered Care Assessment Tool (PCC-AT) in Zambian Health Facilities: Protocol for a Mixed Methods Cross-Sectional Study
Source: JMIR Res Protoc. 2024 Jul 23;13:e54129. doi: 10.2196/54129 (PMC11303880; doi:10.2196/54129)
Supplement: Multimedia Appendix 3 [file resprot_v13i1e54129_app3.docx]

| **SUMMARY OF SCORES BY STANDARD** | | | **ACTION PLAN** | | | | | |
| --- | --- | --- | --- | --- | --- | --- | --- | --- |
| **STAFFING** | | | | | | | | |
| **Subdomain** | **Score** | **Challenge/Issue** | **Potential Actions (please rank them)** | **Priority Action(s) and Selection Justification** | **Expected Output** | **Time-frame** | **Person Responsible** | **Resources needed** |
| Composition |  |  |  |  |  |  |  |  |
| Availability |  |  |  |  |  |  |  |  |
| Competency |  |  |  |  |  |  |  |  |
| Leadership |  |  |  |  |  |  |  |  |
| **SERVICE PROVISION** | | | | | | | | |
| **Subdomain** | **Score** | **Challenge/Issue** | **Potential Actions (please rank them)** | **Priority Action and Selection Justification** | **Expected Output** | **Time-frame** | **Person Responsible** | **Resources needed** |
| Client feedback mechanisms |  |  |  |  |  |  |  |  |
| Service efficiency and Integration |  |  |  |  |  |  |  |  |
| Convenience and access |  |  |  |  |  |  |  |  |
| Digital health worker support tools |  |  |  |  |  |  |  |  |
| **DIRECT CLIENT SUPPORT** | | | | | | | | |
| **Subdomain** | **Score** | **Challenge/Issue** | **Potential Actions (please rank them)** | **Priority Action and Selection Justification** | **Expected Output** | **Time-frame** | **Person Responsible** | **Resources needed** |
| Psychosocial support |  |  |  |  |  |  |  |  |
| Logistical support |  |  |  |  |  |  |  |  |
| Client agency |  |  |  |  |  |  |  |  |
| Digital client support tools |  |  |  |  |  |  |  |  |
